# Tetrandrine Ameliorates Rheumatoid Arthritis in Mice by Alleviating Neutrophil Activities

**DOI:** 10.1155/2022/8589121

**Published:** 2022-02-16

**Authors:** Qingyi Lu, Haixu Jiang, Qingqing Zhu, Jie Xu, Yanan Cai, Guiyang Huo, Kai Yuan, Guangrui Huang, Anlong Xu

**Affiliations:** ^1^School of Life Sciences, Beijing University of Chinese Medicine, Beijing, China; ^2^The Seventh Affiliated Hospital, Sun Yat-sen University, Guangzhou, China; ^3^State Key Laboratory of Biocontrol, Department of Biochemistry, School of Life Sciences, Sun Yat-sen (Zhongshan) University, Guangzhou, Guangdong, China

## Abstract

Rheumatoid arthritis (RA) is a common autoimmune disease worldwide. Neutrophils play critical roles in the onset and development of RA and are the promising target for RA treatment. Tetrandrine is a bis-benzyl isoquinoline alkaloid derived from the traditional Chinese herbal *Stephania tetrandra* S. Moore. Tetrandrine is effective in alleviating RA by inhibiting macrophage inflammatory response, fibroblast overproliferation, and pannus formation. However, whether tetrandrine regulates the activities of neutrophils in RA is largely unknown. In this study, we adopted adjuvant-induced arthritis (AA) murine model to explore the effect of tetrandrine on RA and neutrophils. Twenty-eight mice were divided into four groups. The control group was injected with PBS in the limbs and treated with PBS by intraperitoneal injection (i.p.) from Day 10 to Day 37. The arthritis murine model was induced by injecting FCA into the ankle joints of hind limbs. The AA group, the AA + TET group, and the AA + DEX group mice were treated with PBS, tetrandrine (6 mg/kg), or dexamethasone (1 mg/kg) i.p. daily, respectively. Arthritic scores were evaluated, and the joint diameter was measured every three days. A cytometric bead assay was performed to measure the concentrations of IFN-*γ*, TNF-*α,* and IL-6 in the serum. H&E staining and Safranin O-fast staining were adopted to monitor the tissue changes in the joint. Immunohistochemistry assays were applied to detect the MPO, NE, CitH3, and PAD4 expression levels. To assess the effect of tetrandrine on neutrophil activities *in vitro*, CCK8 tests were applied to determine cell viability. The qPCR and ELISA were performed to determine IL-1*β* and IL-6 expression levels. Immunofluorescence assays were performed to measure the formation of NETs. The results indicated that tetrandrine significantly alleviated the symptoms of RA in terms of the ankle diameter (from 4.629 ± 2.729 to 3.957 ± 0.257; *P* < 0.01) and ankle score (from 4.000 ± 0.000 to 3.286 ± 0.756; *P* < 0.05). Tetrandrine treatment significantly increased the cartilage areas and decreased serum IL-6 significantly (from 5.954 ± 2.127 to 2.882 ± 2.013; *P* < 0.01). The immunohistochemistry assays also showed decreased expression levels of NE, MPO, PAD4, and CitH3 induced by tetrandrine in comparison with the AA group (*P* < 0.01). The qPCR assays and ELISAs showed that tetrandrine had an anti-inflammatory effect *in vitro* by significantly inhibiting IL-6 (*P* < 0.01). The immunofluorescence assays showed that NET formation induced by PMA could be reduced by tetrandrine (*P* < 0.01). In conclusion, tetrandrine has good efficacy in treating RA by regulating neutrophil-involved inflammation and NET formation.

## 1. Introduction

Rheumatoid arthritis (RA) is a common inflammatory disease that results in continuous inflammation, progressive articular damage, and eventually disability. The clinical symptoms include joint stiffness, pain, and swelling.

The onset and progression of RA involve activation of the immune system. Previous studies found that the pathogenesis of RA was correlated with the overresponses of adaptive immunity. However, recent studies have found that innate immunity plays a critical role in the progression of RA with macrophage and neutrophil involvement [1]. In the early stage of RA, neutrophils are activated and recruited to the joint cavity. They continuously secrete chemokines and cytokines, thus maintaining the local inflammatory state [2]. In addition, the specific way that neutrophils defend against the invasion of pathogenic microorganisms is to form neutrophil extracellular traps (NETs) [3], which is called NETosis [1]. This process involves the activation of myeloperoxidase (MPO) [4]. MPO mediates the oxidative activation of neutrophil elastase (NE), which in turn translocates to the nucleus and promotes its proteolysis. In addition, activated peptidyl arginine deiminase 4 (PAD4) participates in the emergence of citrullinated histone H3 (CitH3) by mediating the conversion from arginine to citrulline. With citrullinated histones, nucleic acid depolymerization is triggered [5, 6]. Eventually, intracellular proteins and nucleic acids are released from the cell. The released proteases can cause cartilage proteolysis and bone destruction. Citrullinated proteins can induce the production of anticitrullinated protein antibodies (ACPAs) and other autoantibodies [7]. Therefore, neutrophils can be a primary target for RA treatment strategies [8, 9].

The available treatment options against RA include corticosteroids, disease-modifying anti-rheumatic drugs (DMARDs) (such as Methotrexate), NSAIDs (such as indomethacin), and biologics (such as Infliximab) [10]. However, the long-term application of these anti-inflammatory drugs and immunosuppressants and the excessive use of corticosteroids often result in serious adverse reactions [11–13]. Although biologics have shown outstanding efficacy in the treatment of RA, its drawbacks, including the high expenses, unexpected side effects [14], and the low response of some patients, have motivated scientists and physicians to search for alternative strategies. Fortunately, traditional Chinese medicine (TCM) is complementarily used to ameliorate symptoms and disease progression in both rural and urban areas in China. Therefore, exploring the effect of Chinese herbs in the remission of RA is particularly meaningful in the foreseeable future.

Tetrandrine [(1*β*)-6,6′,7,12-teramethoxy-2,2′-dimethyl-berbaman], known as a bioactive compound derived from the *Stephenia tetrandra* S. Moore dry root, has significant pharmacological effects. In Chinese medicine, *Stephania tetrandra* S. Moore is applied to the treatment of cancer [15], rheumatism, fibrosis, and inflammatory diseases [16]. Experiments have revealed that tetrandrine alleviates arthritis symptoms by inhibiting the migration and invasion of rheumatoid arthritis fibroblast-like synoviocytes [17], inhibiting osteoclastogenesis [18, 19], restoring the Th17/Treg cell balance [20], and regulating macrophage activities [21]. However, research on the impact that tetrandrine exerts on neutrophils is still lacking. Therefore, our study aimed to investigate the effects of tetrandrine on adjuvant-induced arthritis (AA) mice *in vivo* and the neutrophil activities regulated by tetrandrine both i*n vivo* and *in vitro*.

## 2. Materials and Methods

### 2.1. Animals

*C57BL/6 mice* (7-8 weeks old) were purchased from Beijing Vital River Laboratory Animal Technology Company Limited. Specific pathogen-free (SPF) conditions were used to maintain the mice. The animal experiments were authorized by the Beijing University of Chinese Medicine Animal Care and Use Committee (ethics number: BUCM-4-2018060416-2020).

### 2.2. Regents

Lipopolysaccharide (LPS), Freund's complete adjuvant (FCA), tetrandrine, Percoll, and phorbol 12-myristate 13-acetate (PMA) were purchased from Sigma Aldrich (St. Louis, MO, USA). In addition, anti-neutrophil elastase (NE), anti-MPO, anti-CitH3, and anti-*β*-actin antibodies were obtained from Abcam (Cambridge, MA, USA); anti-PAD4 antibody was obtained from ProteinTech Antibody Group (Chicago, IL, USA). Anti-ERK1/2 and anti-phospho-ERK1/2 (Thr202/Tyr204) antibodies were obtained from Cell Signaling Technology (Danvers, MA, USA). HRP-goat anti-rabbit IgG secondary antibody and TRITC-conjugated goat anti-rabbit IgG secondary antibody were purchased from Biodee (Beijing, China). ELISA kits for IL-1*β* and IL-6 were obtained from Invitrogen (Carlsbad, CA, USA). A mouse Th1/Th2/Th17 cytokine kit was obtained from BD Biosciences (Becton, Dickinson and Company).

### 2.3. Adjuvant-Induced Arthritis (AA) Model Induction

Eight-week-old mice were used after one week of adaptive maintenance. The AA model was induced by FCA injection into ankle joints as described previously [22] on Day 0. 20 microlitres of FCA was injected into the ankle cavity, and 80 *μ*L of FCA was injected around the joint. Subsequently, attention was given to the degree of ankle swelling in the mice, and drug intervention was started on Day 3. Joint diameters were evaluated by a pocket thickness gauge (Mitutoyo, Kawasaki, Japan) every three days.

### 2.4. Experimental Design

#### 2.4.1. The Control Group

PBS was injected around the joint on day 0, and intraperitoneal injection of PBS was given beginning on day 3.

#### 2.4.2. The AA Group

CFA was injected around the joint on day 0, and intraperitoneal injection of PBS was given beginning on day 3.

#### 2.4.3. The AA + TET Group

CFA was injected around the joint on day 0, and intraperitoneal injection of tetrandrine (6 mg/kg body weight) was given beginning on day 3.

#### 2.4.4. The AA + DEX Group

CFA was injected around the joint on day 0, and intraperitoneal injection of DEX (1 mg/kg body weight) was given beginning on day 3.

### 2.5. Specimens Collection

After the mice were anesthetized, their eyeballs were removed to collect blood samples. After centrifugation at 3000 rpm for 30 minutes, we collected the supernatant and stored the samples at −80°C until use. The mice were sacrificed by neck dislocation. Samples of ankle tissue were then collected by cutting off the mouse ankles with scissors and removing excessive muscles and skin tissues. After 48 hours in 4% paraformaldehyde solution, the tissues were transferred to 10% EDTA decalcifying solution (pH 7.2–7.4), which was replaced weekly until decalcification was complete.

### 2.6. Histopathology Examination

After decalcification, paraffin sections of the mouse joints were prepared as described previously [22]. HE and Safranin O-fast green staining were conducted using standard procedures. The specific steps were as follows: slices are soaked in xylene for 15 min and repeated twice to complete dewaxing. After dewaxing, the slices were soaked in 100%, 95%, 80%, and 70% ethanol for 5 min. Then, the slices were stained with hematoxylin for 30 seconds and rinsed with running water for 10 minutes. After hematoxylin staining was completed, the slices were stained in eosin solution for 2 min. Then, the slices were soaked in 80%, 95%, and 100% ethanol for 5 min to complete dehydration. Finally, the slices were soaked in xylene for 10 min, repeated twice, and sealed. For Safranin O-fast green staining, the staining time of both Safranin O and fast green was 5 min.

### 2.7. Immunohistochemistry Examination

For immunohistochemistry, the preceding dewaxing and hydration processes were the same as the HE staining steps. After rehydration, the addition of 0.1% Triton X-100 was followed by incubation at 37°C for 20 min. Then, endogenous peroxidase was removed with 3% hydrogen peroxide. The slices were heated in a pressure cooker to complete antigen retrieval, as described in our previous report [22]. The sections were incubated with 10% goat serum at 37°C for 30 min. Primary antibodies at appropriate concentrations were applied. After overnight incubation at 4°C, the corresponding HRP-labelled secondary antibody was incubated at 37°C for 30 min. 3,3′-Diaminobenzidine (DAB) was used to detect positive signals. Hematoxylin was used to stain the nucleus. The following steps were the same as for H&E assays. ImageJ software was used to analyze positive signals.

### 2.8. Neutrophil Preparation and Culture

C57BL/6 mice (7-8 weeks old) were injected with 1 mL 10% protease peptone intraperitoneally, and 1 mL of the solution was injected again after 12 hours. After sacrifice, 5 mL RPMI-1640 medium (containing 10% FBS and 1% antibiotic) was injected into the peritoneal cavity of the mice to obtain lavage fluid. After centrifugation, the cell pellets were resuspended in 1 mL of RPMI-1640 and placed on top of a discontinuous Percoll gradient separation solution (54.8% in the upper layer, 70.2% in the lower layer, 4 mL each). After centrifugation at 500 ×g for 30 minutes, neutrophils were collected at the interface of the upper and lower layers and cultured with different treatments in RPMI-1640 at 37°C in a 5% CO_2_ incubator.

### 2.9. Western Blot

The appropriate number of cells was seeded in a six-well plate. After the addition of LPS or PMA with or without TET, the cells were cultured in a 37°C, 5% CO_2_ incubator for 4 or 5 hours. The protein samples were prepared following standard protocols. Sodium dodecyl sulfate-polyacrylamide gel electrophoresis (SDS-PAGE, 12%) was used to separate the proteins. Then, the protein samples were transferred to PVDF membranes. After incubation with 3% BSA at room temperature for 1 hour, the membrane was incubated with the primary antibodies overnight at 4°C. After washing with TBST, the corresponding HRP-labelled secondary antibodies were incubated with the membrane for 1 h at room temperature. After washing, the membrane was developed in an exposure machine. The greyscale of the band was measured with ImageJ.

### 2.10. Quantitative RT-PCR

TRIzol (Invitrogen, Grand Island, NY, USA) was used to extract RNA from neutrophils. ReverTra Ace qPCR RT Master Mix (Toyobo, Osaka, Japan) was used to perform reverse transcription to cDNA with 1 *μ*g of total RNA. The reaction conditions were 37°C (15 min), 50°C (5 min), 98°C (5 min), and 4°C (hold). The oligonucleotide primer pairs for PCR amplification were listed as follows: *β-actin* (sense: AGAGGGAAATCGTGCGTGAC, antisense: CAATAGTGATGACCTGGCCGT); *IL-6* (sense: CTGCAAGAGACTTCCATCCAG, antisense: AGTGGTATAGACAGGTCTGTTGG); *IFN-γ* (sense: ACAGCAAGGCGAAAAAGGATG, antisense: ACAGCAAGGCGAAAAAGGATG); TNF-*α* (sense: ACAGAAAGCATGATCCGCG, antisense: GCCCCCCATCTTTTGGG); and *IL-1β* (sense: AGTTCCCCAACTGGTACATCAG, antisense: TCAATTATGTCCTGACCACTGTTG). SYBR Green Real-time PCR Master Mix was used to conduct qRT-PCR (Toyobo, Osaka, Japan). The reaction conditions were as follows: denaturation at 95°C (15 s) and annealing extension at 60°C (1 min). The above conditions require 35–44 cycles. The specificity of amplified PCR products was evaluated by melting curve analysis. Relative expression levels were evaluated with the 2^−ΔΔCt^ method. The fold changes in gene expression were normalized to *β*-actin levels.

### 2.11. Inflammatory Factors Detection with a CBA Kit

A cytometric bead array (CBA) kit was used to detect the inflammatory factors in the serum. The experimental procedures were determined according to the manufacturer's instructions. In brief, 50 *μ*L of cytokine capture magnetic beads was mixed evenly and added to the sample and standard. After adding 50 *μ*L detection reagents, the tubes were incubated for 2 hours in the dark at room temperature. After 3 washes, the signals were detected by flow cytometry and evaluated with FCAP Array software.

### 2.12. Inflammatory Factors in Cell Culture Supernatant Detection with ELISA Kits

Cytokines in the mouse neutrophil culture supernatant were evaluated by enzyme-linked immunosorbent assay (ELISA) kits, and the specific experimental procedures followed the manufacturer's instructions. The culture medium was collected after treatment. The supernatant was collected by centrifugation. All of the following steps were implemented according to the instructions. The specific procedure was as follows: 100 *μ*L of sample diluent buffer was added to one well as a negative control, and 100 *μ*L diluted standard or sample was added to the remaining wells. Then, 100 *μ*L of detection antibody was added for incubation at 37°C for 1 h, followed by 100 *μ*L HRP-labeled antibody for incubation at 37°C for 1 h. Then, 100 *μ*L TMB color solution was added to each well, and the color (blue) was developed at 37°C for 5 min. After 100 *μ*L stop solution was added, the blue color turned yellow. Finally, the optical density (OD) of each well was measured at 450 nm with a microplate reader. The concentration of cytokines was calculated accordingly.

### 2.13. Immunofluorescence Staining

After stimulation for 4 hours, 4% paraformaldehyde was used to fix the neutrophils. Then, the cells were ruptured by 0.1% Triton X-100 and immersed in 5% BSA at room temperature for approximately 45 min for blocking. The cells were incubated with a primary antibody of the appropriate concentration for the target protein at 4°C. After overnight incubation, the secondary antibody was applied and the nuclei were stained with DAPI. A laser confocal microscope was used to evaluate the fluorescence signal. The staining intensity was measured and recorded using ImageJ software.

### 2.14. Statistical Analysis

The mean ± standard deviation (SD) was used to express the data. *T*-tests and one-way analysis of variance were also conducted. *P* < 0.05 was considered significant.

## 3. Results

### 3.1. Tetrandrine Suppressed Inflammation in Adjuvant-Induced Arthritis Mice

**Figure 1:**
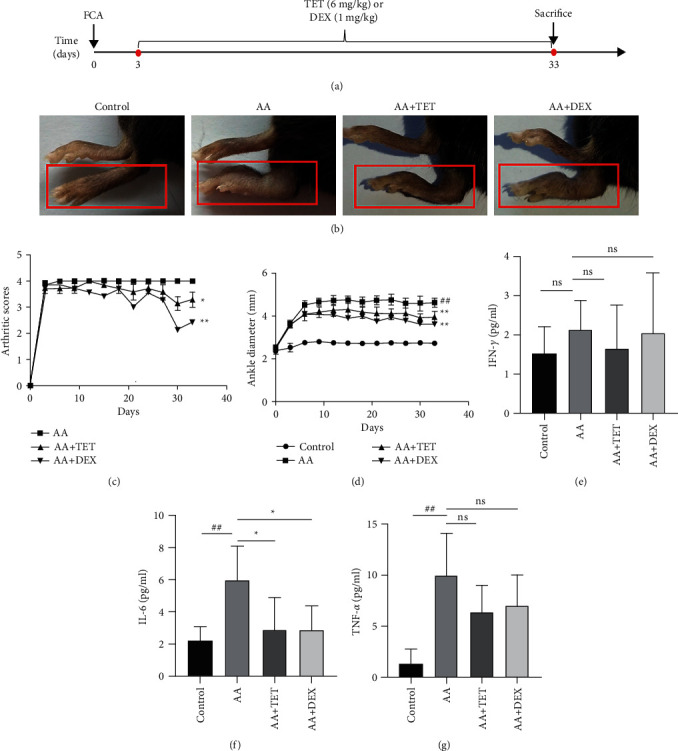
The effect of TET on the AA mouse model. AA mice were injected with PBS, TET, and DEX intraperitoneally every day. The joint data were acquired every three days. The sera were acquired after sacrifice. (a) The schematic diagram of the mice experiment. AA inflammation was induced on Day 0, and the drugs were given beginning on Day 3. (b) Representative images of the paws of the mice. The limbs that were injected with PBS or FCA are marked by the red rectangle. (c) The arthritic score of the AA mice. (d) The ankle joint diameter of the mice. (e) The concentration of IFN-*γ* in each group. (f) The concentration of IL-6 in each group. (g) The concentration of TNF-*α* in each group. For each group, *n* ≥ 5, the bars are represented by the mean ± SD ^##^*P* < 0.01 compared with the control group.  ^*∗*^ ^*∗*^*P* < 0.01 compared with the AA group.  ^*∗*^*P* < 0.05 compared with the AA group. Ns, *P* > 0.05, the difference was statistically nonsignificant.

Upon induction, the ankle diameter and inflammatory scores were assessed every three days (Figure 1(a)). The pictures of the injected limb at the end of the experiment directly indicated the efficacy of TET (Figure 1(b)). As Figure 1(c) shows, on Day 33, TET (3.286 ± 0.756; *P* < 0.05) and DEX (2.429 ± 0.535; *P* < 0.01) significantly decreased the ankle scores in comparison with that of the AA group (4.000 ± 0.000), while the scores of the control group were set as 0.  Figure 1(d) describes the ankle diameter of the different groups. On Day 33, the diameter of the AA group was much greater than that of the control group (4.629 ± 2.729 vs. 2.729 ± 0.023; *P* < 0.01). TET (3.957 ± 0.257; *P* < 0.01) and DEX (3.629 ± 0.152; *P* < 0.01) reduced the ankle diameters. Additionally, the cytokine concentration was determined to reflect the overall degree of inflammation (Table 1 and Figures 1(e)–1(g)). Tetrandrine greatly inhibited IL-6 (Figure 1(f)) secretion.

**Figure 2:**
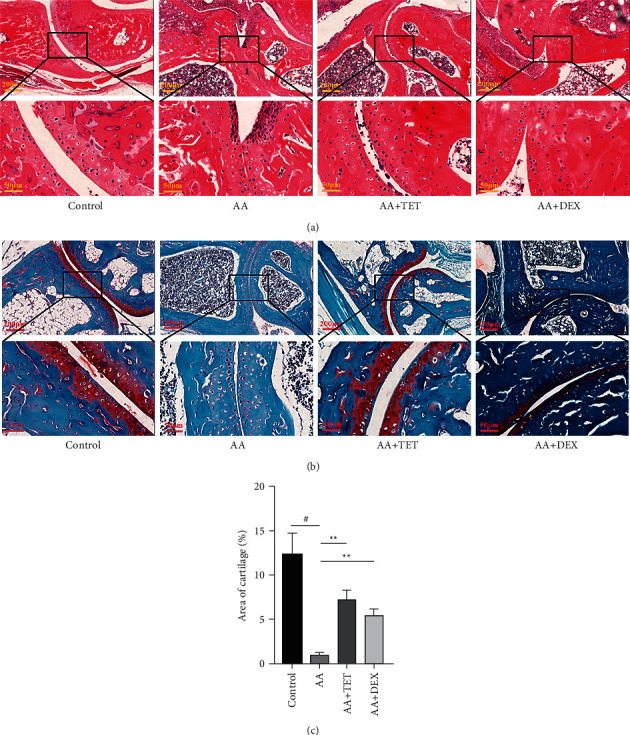
The histopathological changes by TET in the AA mice. (a) Hematoxylin and eosin staining was applied to assess the basic histopathological changes. P: the pannus; 1: reduced joint cavity; 2: bone destruction. (b) Safranin O-fast green staining was applied to assess articular cartilage damage. The red color indicates cartilage tissue. Images of representative sections from three groups are shown. (c) Quantification of cartilage areas was analyzed. ^#^*P* < 0.05 compared with the control group.  ^*∗*^ ^*∗*^*P* < 0.01 compared with the AA group.

Furthermore, H&E staining (Figure 2(a)) revealed basic pathological changes. Safranin O-fast green staining (Figures 2(b) and 2(c)) focused on the bone protective function of tetrandrine. The bone erosion of the AA group was worse than that of the control group (0.951 ± 0.169 vs. 12.23 ± 1.424; *P* < 0.05). TET (7.174 ± 0.642; *P* < 0.01) and DEX (5.397 ± 0.445; *P* < 0.01) attenuated bone destruction.

### 3.2. Tetrandrine Inhibited Neutrophil Infiltration and Activation in AA Mice

**Figure 3:**
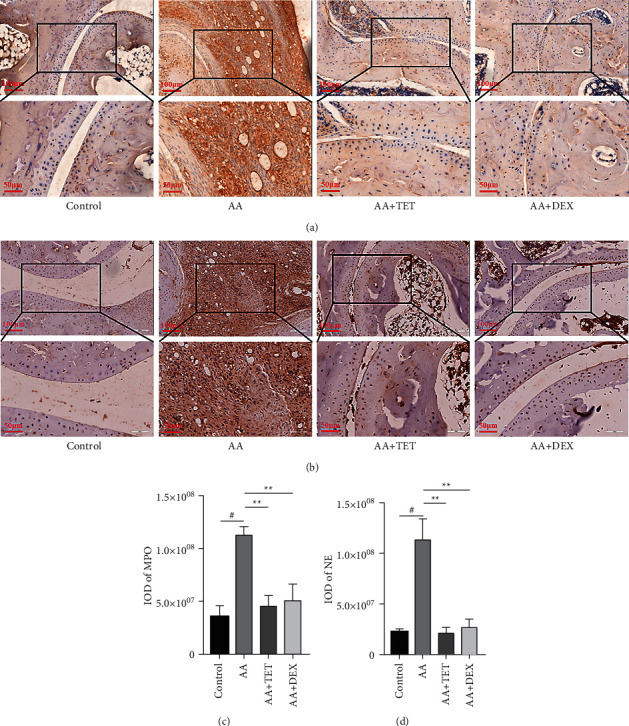
The effect of TET on MPO and NE expression in ankle joint tissue of the AA mice. Dark brown indicates the specific protein expression. (a) The expression of MPO in ankle joint tissues. (b) The expression of NE in local joint tissues. (c) The dark areas that indicate MPO expression were analyzed and presented as the mean ± SD (d) The dark areas that indicate NE expression were analyzed and presented as the mean ± SD. Images of representative sections from three groups are shown. ^#^*P* < 0.05 compared with the control group.  ^*∗*^ ^*∗*^*P* < 0.01 compared with the AA group.

To investigate the infiltration of neutrophils, immunohistochemistry assays were applied to detect the MPO (Figure 3(a)) and NE (Figure 3(b)) expression levels. Their quantifications were analyzed (Figures 3(c) and 3(d), Table 2). MPO and NE were significantly decreased by tetrandrine and DEX compared with the AA group (*P* < 0.01).

**Figure 4:**
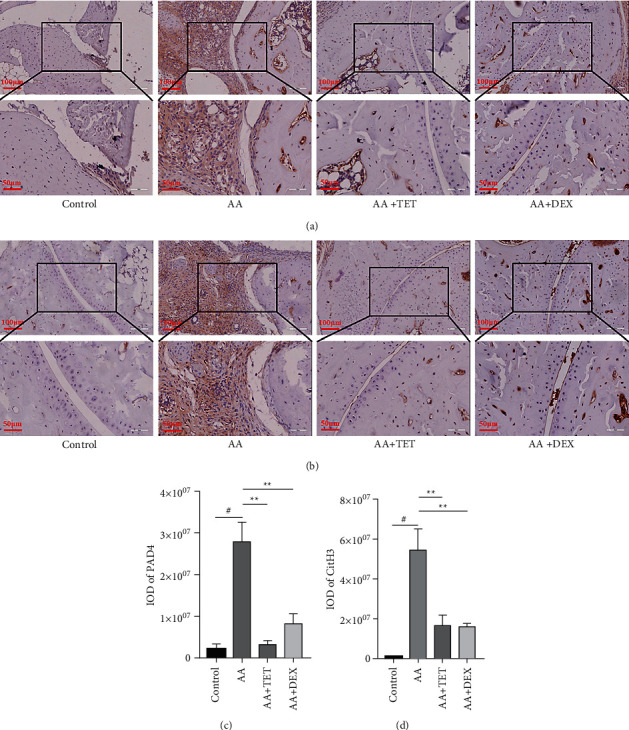
The effect of TET on NETs formation *in vivo*. (a) The effect of TET on PAD4 expression in the ankle joint tissue of the AA mice. (b) The effect of TET on CitH3 expression in the ankle joint tissue of the AA mice. (c) Quantification of dark brown areas indicating PAD4 expression. (d) Quantification of dark brown areas indicating CitH3 expression. Images of representative sections from the three groups are shown. ^#^*P* < 0.05 compared with the control group.  ^*∗*^ ^*∗*^*P* < 0.01 compared with the AA group.

PAD4 (Figure 4(a)) and CitH3 (Figure 4(b)) were detected, and the dark brown areas were analyzed (Figures 4(c) and 4(d), Table 2). The results indicated that the increased PAD4 expression in the mice of the AA group could be inhibited by tetrandrine (*P* < 0.01), indicating that tetrandrine reduced NET formation.

### 3.3. Tetrandrine Suppressed LPS-Induced Proinflammatory Activities *In Vitro*

**Figure 5:**
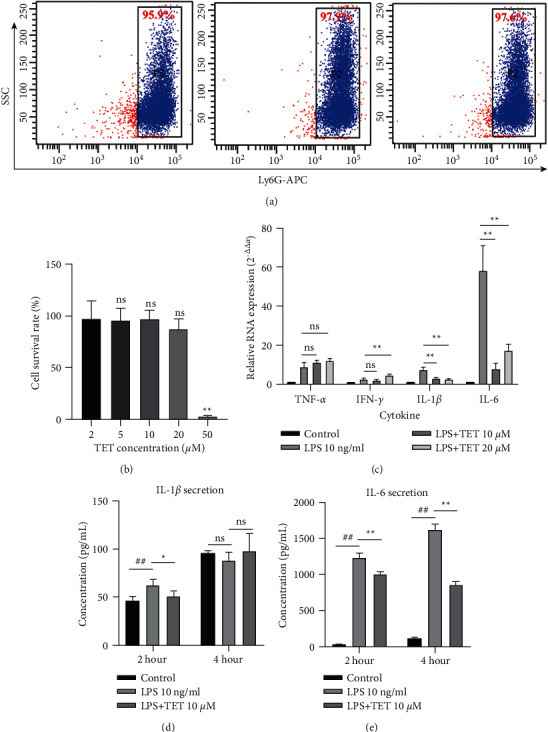
Tetrandrine inhibited cytokine secretion by neutrophils *in vitro*. (a) FACS tests for Ly6G-positive cells after purification of peritoneal neutrophils. (b) The effect of TET on the viability of neutrophils. A CCK-8 assay was performed to assess neutrophil viability after four hours of coculture with different concentrations of tetrandrine. For each group, *n* = 5. (c) The effect of TET on neutrophil TNF-*α*, IFN-*γ*, IL-1*β,* and IL-6 mRNA by qRT-PCR. For each group, *n* = 3. (d) The concentration of IL-1*β* after two-hour and four-hour LPS culture with or without TET. (e) The concentration of IL-6 after two-hour and four-hour LPS culture with or without TET. The results are presented as mean ± SD ^##^*P* < 0.01 compared with the control group.  ^*∗*^*P* < 0.05 compared with the LPS group.  ^*∗*^ ^*∗*^*P* < 0.01 compared with the LPS group.

To determine the mechanism by which tetrandrine addresses the inflammatory circumstances, LPS was utilized to induce the inflammatory activities of neutrophils *in vitro*, and cytokine secretion was assessed. Next, neutrophils were purified via peritoneal injection, and the purity was authorized by FACS by marking Ly6G, which was generally over 95% (Figure 5(a)). A suitable concentration that would not affect neutrophil viability was explored via the CCK-8 assay (Figure 5(b)). Cell survival rates were calculated on the basis of the blank group into which no TET was added. TET at 2 *μ*Μ (96.79 ± 17.90; *P* > 0.05), 5 *μ*Μ (95.88 ± 11.96; *P* > 0.05), 10 *μ*Μ (97.12 ± 8.621; *P* > 0.05), and 20 *μ*Μ (87.35 ± 9.783; *P* > 0.05) maintained cell viability, while 50 *μ*Μ (3.182 ± 0.5545; *P* < 0.01) TET significantly affected the survival of the cells.

Then, the transcription level of the cytokine genes was measured by qPCR (Figure 5(c), Table 3), which indicated that 10 *μ*Μ tetrandrine significantly inhibited *IL-6* and *IL-1β* transcription (*P* < 0.01). We adopted 10 *μ*Μ for the following *in vitro* assays. ELISAs (Table 4) showed that after a two-hour incubation, TET decreased IL-1*β* (Figure 5(d), *P* < 0.05) and IL-6 (Figure 5(e), *P* < 0.01). TET suppressed IL-6 after the four-hour incubation (Figure 5(e), *P* < 0.01).

**Figure 6:**
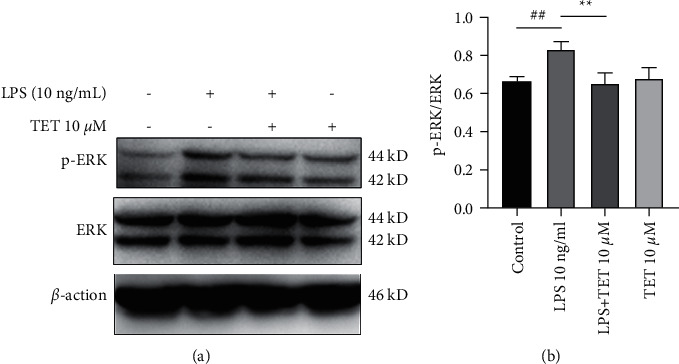
Tetrandrine inhibited the phosphorylation of ERK in neutrophils *in vitro*. (a) Western blot results of p-ERK/ERK in cells with different treatments for 4 h. (b) Density analysis of p-ERK/ERK in (a). The results are presented as mean ± SD ^##^*P* < 0.01 compared with the control group.  ^*∗*^ ^*∗*^*P* < 0.01 compared with the LPS group.

To explore the anti-inflammatory mechanism, the expression levels of p-ERK/ERK involved in MAPK pathways were detected via WB and quantified (Table 5). The results showed that tetrandrine aggressively decreased the phosphorylation of ERK (Figure 6) (*P* < 0.01).

### 3.4. Tetrandrine Inhibited NET Formation *In Vitro*

**Figure 7:**
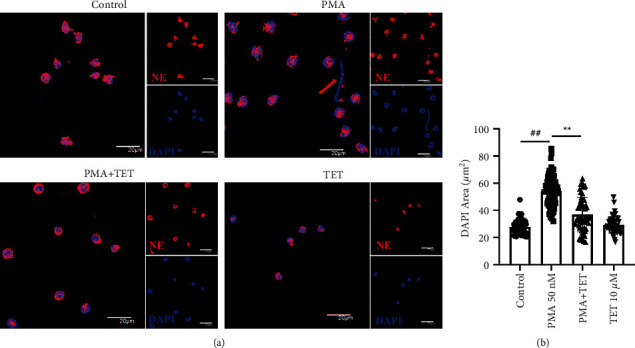
The effect of TET on NETs formation *in vitro*. After stimulation with PMA for 5 h, neutrophils were stained with DAPI (blue) and anti-NE antibody (red) to visualize NETs using confocal microscopy. (a) NETs were formed after PMA stimulation. TET inhibited the NETs formation. (b) Nuclear areas were quantified and analyzed. Tetrandrine decreased the enlargement of the nuclear areas induced by PMA. Values are presented as mean ± SD ^##^*P* < 0.01 compared with the control group.  ^*∗*^ ^*∗*^*P* < 0.01 compared with the PMA group.

PMA was applied to activate NET formation in the immunofluorescence assay. In neutrophils, DNA was stained with DAPI in blue, and NE was stained with a red fluorescent marker. During NET formation, nuclear areas were enlarged (54.19 ± 11.52 vs. 27.71 ± 5.124; *P* < 0.01), and DNA overflowed out of the cell (red arrow in Figure 7(a)). Tetrandrine inhibited chromatin decondensation (Figure 7(a)). TET decreased the nuclear size induced by PMA (36.99 ± 12.75; *P* < 0.01), while TET alone (29.39 ± 5.984) had no effect (Figure 7(b)).

## 4. Discussion

TCM is widely applied in RA treatment due to its low cost and high safety. We consider tetrandrine to be a promising natural compound that could be a new treatment strategy. An adjuvant-induced arthritis model was established because it is similar to arthritis in humans.

In our experiments, TET decreased arthritic scores and joint diameter. The volume of the paws should have been tested to strengthen our evidence [23]. H&E assays confirmed the efficacy of TET. The amelioration of joint inflammation corresponded with the reduction of IL-6 levels in the serum. Compared with the control group, the AA group had significant increases in TNF-*α* and IL-6, which could be decreased by tetrandrine, especially IL-6. It was previously found that tetrandrine inhibited TNF-*α* and IL-1*β* in FCA-induced arthritis at a concentration of 20 mg/kg [24].

To assess the infiltration and activation of neutrophils, IHC assays were performed. MPO is a peroxidase mainly expressed in neutrophils [25]. NE is associated with the destruction of cartilage [26]. Based on these findings, we detected the expression of MPO and NE to track the infiltration of neutrophils. Meanwhile, the expression of CitH3 and PAD4 was also measured. Previous studies showed that anti-PAD4 autoantibodies could be used as biomarkers in early RA patients [27]. PAD4 is considered indispensable for NET formation [28]. It was shown that pad4^−/−^ neutrophils failed to form NETs after being stimulated by chemokines or incubated with bacteria [29]. CitH3 produced by PAD4 is recognized as an autoantigen by the host. Our results showed that TET not only inhibited infiltration in mice but also suppressed the formation of NETs.

Our RT-PCR and ELISAs showed that tetrandrine inhibited IL-6 and IL-1*β in vitro*. IL-6 was an important inflammatory cytokine. It can induce VEGF production, resulting in excessive angiogenesis, vascular permeability, bone resorption, and osteoporosis [30]. IL-6 has an additional effect on the acute phase response of RA, which can trigger a systemic inflammatory response [31]. Thus, aberrant production of IL-6 can cause systematic symptoms [32]. Our results also revealed the suppressive impact of TET on IL-1*β* secretion within a two-hour incubation *in vitro*. It was previously reported that TET suppressed the level of IL-1*β* in macrophages induced by LPS [24] or *β*-glucan [21]. IL-1 can cause cartilage damage and bone resorption. However, IL-1*β* is not considered to be the leading cytokine in the pathogenesis of RA because inhibition of IL-1 failed to achieve a curative effect [33, 34].

MAPK is responsible for the expression of proinflammatory cytokines upon LPS activation. Tetrandrine was proven to inhibit the phosphorylation of JNK in MH7A cells [17]. The phosphorylation of ERK was inhibited by TET in macrophages [21]. Our data showed that tetrandrine reduced the phosphorylation of ERK in neutrophils, which indicated that MAPK signaling may be the mechanism by which tetrandrine regulates IL-6 secretion.

Our *in vivo* results suggested that the formation of NETs was inhibited in mice by tetrandrine. The immunofluorescence assay provided *in vitro* evidence that tetrandrine decreased the nuclear sizes and maintained DNA within the cells. During NETosis, DNA flows out of the cells, accompanied by the release of intracellular proteases. Among them, NE is a serine protease. It plays a key role in promoting inflammatory responses. It catalyzes the breakdown of extracellular matrix proteins. Based on our results, inhibiting NETs could be one of the ways that tetrandrine alleviated arthritis.

## 5. Conclusion

Our study clarified the effect of tetrandrine on AA mice and neutrophils. Tetrandrine alleviated joint edema in FCA-induced arthritis and suppressed the infiltration and activation of neutrophils *in vivo*. Its inhibitory effect on IL-6 may be related to reducing the phosphorylation of ERK. In addition, tetrandrine reduced NETs formation. However, whether decreased NETs are associated with the alleviation of arthritis remains unknown. Determining the role that NETs play in RA requires further research. In conclusion, our study found that tetrandrine might be a promising therapeutic agent in the treatment of RA.

## Figures and Tables

**Table 1:** TET attenuated inflammatory cytokines in the serum.

Cytokines	The control group	The AA group	The AA + TET group	The AA + DEX group
IFN-*γ*	1.518 ± 0.692	2.139 ± 0.740	1.650 ± 1.114	2.053 ± 1.525
IL-6	2.192 ± 0.887	5.954 ± 2.127	2.882 ± 2.013	2.873 ± 1.504
TNF-*α*	1.290 ± 1.450	9.984 ± 4.126	6.403 ± 2.589	7.018 ± 3.002

Values are denoted as the mean ± SD

**Table 2:** TET regulated neutrophil recruitment and activation *in vivo*.

Markers	The control group (×10^7^)	The AA group (×10^7^)	The AA + TET group (×10^7^)	The AA + DEX group (×10^7^)
MPO	3.558 ± 0.598	11.217 ± 0.497	4.541 ± 0.577	5.060 ± 0.911
NE	2.216 ± 0.178	11.338 ± 1.194	2.121 ± 0.322	2.685 ± 0.484
PAD4	0.207 ± 0.076	2.784 ± 0.271	0.327 ± 0.052	0.822 ± 0.138
CitH3	0.080 ± 0.018	5.444 ± 0.615	1.646 ± 0.308	1.604 ± 0.094

The results are presented as mean ± SD

**Table 3:** TET reduced the mRNA levels of IL-1*β* and IL-6.

Markers	Control	LPS	LPS + TET 10 *μ*Μ	LPS + TET 20 *μ*Μ
TNF-*α*	1.000 ± 0.000	8.7531 ± 2.508	11.07 ± 1.280	11.89 ± 1.333
IFN-*γ*	1.000 ± 0.000	2.367 ± 0.810	1.943 ± 0.534	4.473 ± 0.669
IL-1*β*	1.000 ± 0.000	7.513 ± 1.113	2.893 ± 0.573	2.540 ± 0.292
IL-6	1.000 ± 0.000	58.000 ± 13.000	7.890 ± 2.790	17.050 ± 3.388

The results are presented as mean ± SD. The control group was set to 1.000 ± 0.000.

**Table 4:** TET reduced the secretion of IL-1*β* and IL-6.

Markers	Control	LPS	LPS + TET 10 *μ*Μ
IL-1*β* 2 h	46.390 ± 4.349	62.440 ± 6.224	51.390 ± 5.350
IL-1*β* 4 h	95.850 ± 2.485	87.870 ± 8.930	98.140 ± 18.010
IL-6 2 h	36.180 ± 2.143	1229.000 ± 66.800	1005.000 ± 33.890
IL-6 4 h	113.000 ± 15.64	1618.000 ± 78.83	856.100 ± 45.200

The results are presented as mean ± SD

**Table 5:** TET inhibited phosphorylation of ERK.

Markers	Control	LPS	LPS + TET	TET
p-ERK/ERK	0.663 ± 0.025	0.831 ± 0.041	0.651 ± 0.058	0.678 ± 0.058

The results are presented as mean ± SD

## Data Availability

Data are available on request.
